# The Dark Side of Malleability: Incremental Theory Promotes Immoral Behaviors

**DOI:** 10.3389/fpsyg.2017.01341

**Published:** 2017-08-07

**Authors:** Niwen Huang, Shijiang Zuo, Fang Wang, Pan Cai, Fengxiang Wang

**Affiliations:** ^1^Faculty of Psychology, Beijing Normal University Beijing, China; ^2^Beijing Key Lab of Applied Experimental Psychology Beijing, China; ^3^National Demonstration Center for Experimental Psychology Education, Beijing Normal University Beijing, China; ^4^The General Hospital of the PLA Rocket Force Beijing, China

**Keywords:** implicit theories, incremental theory, entity theory, immoral behavior, moral decisions, fundamental attribution error

## Abstract

Implicit theories drastically affect an individual’s processing of social information, decision making, and action. The present research focuses on whether individuals who hold the implicit belief that people’s moral character is fixed (entity theorists) and individuals who hold the implicit belief that people’s moral character is malleable (incremental theorists) make different choices when facing a moral decision. Incremental theorists are less likely to make the fundamental attribution error (FAE), rarely make moral judgment based on traits and show more tolerance to immorality, relative to entity theorists, which might decrease the possibility of undermining the self-image when they engage in immoral behaviors, and thus we posit that incremental beliefs facilitate immorality. Four studies were conducted to explore the effect of these two types of implicit theories on immoral intention or practice. The association between implicit theories and immoral behavior was preliminarily examined from the observer perspective in Study 1, and the results showed that people tended to associate immoral behaviors (including everyday immoral intention and environmental destruction) with an incremental theorist rather than an entity theorist. Then, the relationship was further replicated from the actor perspective in Studies 2–4. In Study 2, implicit theories, which were measured, positively predicted the degree of discrimination against carriers of the hepatitis B virus. In Study 3, implicit theories were primed through reading articles, and the participants in the incremental condition showed more cheating than those in the entity condition. In Study 4, implicit theories were primed through a new manipulation, and the participants in the unstable condition (primed incremental theory) showed more discrimination than those in the other three conditions. Taken together, the results of our four studies were consistent with our hypotheses.

## Introduction

Implicit theories have enjoyed several decades of empirical interest and support. These theories pertain to the nature of human attributes and propose that individuals have different epistemological understandings of the world (Dweck et al., 1995a; Molden and Dweck, 2006). In general, there are two types of implicit theories: incremental theory and entity theory (Dweck and Leggett, 1988; Hong et al., 1997, 1999). Entity theorists believe that one’s personal characteristics are decided by inheritance and innateness and are thus fixed and unchangeable (Dweck et al., 1993; Hughes, 2015). By contrast, incremental theorists believe that characteristics are shaped by the environment and personal growth and are thus malleable and changeable (Chiu et al., 1997b; Hughes, 2015). People can hold different theories in different domains (e.g., intelligence, personality). Myriad research (see the review by Dweck, 1999; Burnette et al., 2013) has shown that implicit theories create a framework for people to understand the world (Dweck et al., 1995a) and have a broad impact on individuals’ social judgment, intergroup perceptions, personal development, and self-regulation. The present research focuses on implicit theories about people’s moral character. Specifically, we seek to examine how individuals’ implicit theories about whether moral character is fixed or changeable (Chiu et al., 1997a) shape their own moral decisions.

According to the existing findings, a remarkable feature of implicit theories is that incremental theory brings about more social beneficial outcomes than entity theory. Different evidence has indicated that compared to entity theorists, incremental theorists obtain better academic performance (Blackwell et al., 2007), overcome personal setbacks more easily (Burnette and Finkel, 2012), and endorse less extreme stereotypes (Levy et al., 1998). In the present study, however, we argue that incremental theory also has its drawbacks when applied to moral decisions.

First, previous research has identified how two different implicit theories predict the degree to which people make behavior inferences from the limited information of target individuals. Specifically, incremental theorists who believe that one’s behaviors vary across situations rather than being fixed (Dweck et al., 1993, 1995a; Levy and Dweck, 1998) are more likely to infer people’s behavior from situational factors and are less likely to expect behavior to be highly consistent and highly predictive. They are thus less likely to view a concrete behavior as a manifestation of an underlying trait or even to view traits as the most useful method of characterizing individuals (Dweck et al., 1995a; Chiu et al., 1997b; Gervey et al., 1999; Wurthmann, 2013). By contrast, entity theorists make more trait attributions and pay more attention to information consistent with stereotypes (Plaks et al., 2001, 2005). Entity theorists believe that traits are the main cause of behavior and have greater predictive value than situational factors and, therefore, that a small sample of behavior will provide a reliable reading of a trait (Chiu et al., 1997b; Tam et al., 2010). In addition, incremental theorists and entity theorists have a different tendency toward making the fundamental attribution error (FAE), which described the general tendency to overestimate the importance of dispositional factors relative to environmental influences (Ross, 1977). Incremental theorists are more likely to attribute people’s moral decision and performance to the situational factors rather than their personalities (e.g., Dweck et al., 1995a), which means they are less likely to make the FAE; whereas, entity theorists rely more on traits rather than situational factors (e.g., Plaks et al., 2001), which is more consistent with the view of the FAE. This difference may be one of the reasons why they make different choices when facing a moral decision.

Second, prior studies have found that incremental theorists show higher tolerance to other people’s immoral behaviors than entity theorists. For instance, incremental theorists made fewer negative evaluations, showed more empathy, and recommended less punishment to an immoral target (Erdley and Dweck, 1993; Gervey et al., 1999), because they believed that human actions were dynamic and malleable, and thus the problem behaviors could be educated or reformed (Kammrath and Peetz, 2012). By contrast, entity theorists had a greater tendency to support rigid punishment and to show more negative attitudes toward moral transgressions (Chiu et al., 1997a; Miller et al., 2007; Tam et al., 2013), because they believed that traits were fixed and essential, and thus those who had once offended moral principles were very likely to recidivate (Tam et al., 2013; Williams, 2015). Furthermore, incremental theorists were more likely to forgive and trust after an apology than entity theorists (Haselhuhn et al., 2010). Incremental theorists’ empathy and inclusion seem to be socially appreciated; however, there is typically a highly positive correlation between the tolerance of immoral behavior committed by others and by oneself (Wang and Sun, 2016). Therefore, it is likely that incremental theorists show a high tolerance for their own immoral behaviors as well.

In moral decision making, people typically weigh the impact of the subsequent behavior on their moral self-image (Mazar et al., 2008). Individuals always expect to maintain a positive self-image (Jones, 1973; Monin and Jordan, 2009), and without question, to engage in immoral behavior means to damage one’s moral self-image to some degree (Jordan et al., 2015). For entity theorists, the damage could be fatal since they are more prone to make the FAE, give a trait explanation for behaviors and make moral judgments basing on limited and concrete information; more importantly, they tend to not forgive transgressions. As a result, to maintain a positive moral self-image, entity theorists will have a low intention to engage in immoral behaviors. However, it is a different story for incremental theorists. They are less likely to make the FAE because they tend to explain behaviors in terms of malleable context information; a single immoral behavior will not make them waver in their trust in their moral self-image. Furthermore, even if the moral self-image has been harmed to some extent because of the current immoral behavior, they could do something such as apologize or commit to change to remedy. Therefore, there might be less inner constraint in incremental theorists than in entity theorists when engaging in immoral behaviors.

Previous studies referring to implicit theories of morality shed light on how individuals’ implicit beliefs influence their attitudes toward and judgments of other people, such as punishments for criminals (Tam et al., 2013) and forgiveness for an immoral target (Haselhuhn et al., 2010). However, very little attention has been paid to how implicit theories shape one’s own moral decisions. This is a very important question, and answering it would help us understand the process of people’s moral decision making. Therefore, the present study focuses on the influence of implicit theories on one’s own moral performance. In addition, previous studies about implicit theories revealed that incremental theorists were less likely to make the FAE, showed lower tendency to make moral judgments through limited information and had higher tolerance toward one’s immoral actions (e.g., Dweck et al., 1995a; Gervey et al., 1999), relative to entity theorists. These characteristics of incremental theorists may strengthen their ability to maintain a self-image and reduce the disgust in response to immorality. Therefore, these characteristics may reduce the costs associated with engaging in immoral behaviors, suggesting the underlying dark side of incremental theory.

Accordingly, we posit that an incremental (not entity) moral belief could facilitate one’s immoral behavior. However, this view is very different from previous ones in the field of implicit theories and there was little direct and conclusive evidence that supports this novel viewpoint, so we did not make a strong hypothesis at the very beginning. Instead, we would preliminarily explore the relation between implicit theories of morality and immoral behavior with an open-ended prediction in Study 1 and then clarify our hypothesis in the subsequent studies according to the results of Study 1. Four studies in total were conducted to explore whether individuals who possess an incremental or an entity moral belief show different behaviors when facing a choice between moral and immoral behaviors. Specifically, in Study 1, the relation between implicit theories of morality and immoral behavior was explored from the perspective of observers. In Studies 2–4, we further replicated the observed relationship from the actor perspective. Study 2 utilized self-report measures of incremental and entity moral theories to examine the connection of implicit theories and discrimination behavior in a hiring situation. In Study 3, we primed the two different implicit theories since they can display either relatively stable trait-like or temporary mental representation (Leith et al., 2014) and observed how they behave in the following task which involved the possibility of cheating for self-interest. In Study 4, implicit theories of morality were further distinguished into three types: unstable, stable and moral, and stable and immoral; we manipulated these types of beliefs and explored their impacts on the participants’ subsequent act of discrimination.

## Study 1

Study 1 was designed to preliminarily examine the association between implicit theories and immoral behavior from the perspective of observers. In this study, the participants were introduced to two people with different implicit theories and were then required to judge which person would be more likely to behave immorally. One possible outcome of this study is that entity theory will be connected to immorality, because previous research found the relationship between entity theory and negative implications like endorsing stereotypes (e.g., Levy et al., 1998). However, another possibility is that incremental theory will be related to immorality, because incremental theorists are less likely to make the FAE, rarely depend on limited information to presume one’s traits and have a high tolerance to immorality (e.g., Dweck et al., 1995a; Gervey et al., 1999), which enables to maintain their bright self-images when they engage in immoral behavior.

### Method

#### Participants

One hundred and ninety-three participants were recruited to complete a questionnaire online in exchange for ¥6 Yuan (approximately $0.9 USD). Twenty-three participants were excluded because they failed to answer the questions correctly at the beginning of the task in this study. Ultimately, 170 participants (44 males, 126 females) were included in the analysis. The ages ranged from 17 to 44, with a mean of 24.58 (*SD* = 4.78).

#### Materials and Procedure

The participants were first presented a description of two people (*A* and *B*) with different implicit theories of morality. *A* was described as follows: “*A believes that one’s morality is relatively stable… He thinks that our morality has been determined at the earliest of childhood and is mainly determined by genetic factors. Society, education, and some other environmental factors have almost no influence on morality*” (i.e., an entity theorist); whereas *B* was described as follows: “*B believes that one’s morality is relatively changeable…He thinks that our morality will change with a different environment. Society, education, and some other environmental factors have an important influence on the morality, but genetic component seems to have minimal influence*” (i.e., an incremental theorist; for the full descriptions, see the **Appendix**). The participants were required to remember the characteristics of *A* and *B* and answer four questions about them (e.g., who believes morality is relatively stable?). Only the participants who answered all four questions correctly could continue to complete the subsequent tasks. Next, the participants were instructed to judge the possible performance of *A* and *B* in a series of everyday life behaviors (*Everyday Immoral Activity List*) and an environmental destruction situation (*Forest-Management Scene*). Finally, the participants reported their demographic information.

##### Everyday immoral activity list

An Everyday Immoral Activity List was developed to measure the likelihood of behaving immorally in everyday life. The list consists of 10 common immoral behaviors (e.g., “Use unfair means to obtain a better grade on the examination or quiz”; for the full items, see the **Appendix**). The participants rated the likelihood of performing such immoral behaviors between *A* and *B* on a seven-point scale (1 = *A more likely*, 4 = *equally between A and B*, 7 = *B more likely*). The Cronbach’s α = 0.90.

##### Forest-management scene

A Forest-Management Scene was adapted from Kasser and Sheldon (2000) to measure the likelihood of damaging the environment to gain benefits. The scene described that *A* and *B* each owned a company that had to bid against three other companies to harvest timber within the same national forest. *A* and *B* both knew that the forest might disappear if their companies continually made large bids. The participants were asked to rate the likelihood of profiting more than the other companies, cutting at a faster rate, and appealing to the other three companies to reduce their cutting between *A* and *B* on a seven-point scale (1 = *A more likely*, 4 = *equally between A and B*, 7 = *B more likely*). The third item were reverse scored, and the Cronbach’s α = 0.82.

### Results

We conducted one-sample *t*-tests to examine the difference in behaving immorally between *A* and *B*, as rated by the participants. The results showed that the score of the Everyday Immoral Activity List was significantly higher than the midpoint 4 (*M* = 4.90, *SD* = 1.28; *t*(169) = 9.14, *p* < 0.001, 95% CI [0.70, 1.09], Cohen’s *d* = 0.703), which means that the participants think that *B* was more likely to behave immorally in everyday life. Similarly, the score of the Forest-Management Scene was also significantly higher than the midpoint 4 (*M* = 4.99, *SD* = 1.64; *t*(169) = 7.19, *p* < 0.001, 95% CI [0.75, 1.24], Cohen’s *d* = 0.604), which means that the participants think that *B* was more likely to damage the environment to gain benefits.

We performed a sensitivity analysis using G^∗^Power 3.1 (Faul et al., 2009) to estimate the effect size that the study was able to detect with a power of 80% (Cohen, 1992) and α = 0.05. The results indicate that the minimum effect size to which the one-sample *t*-tests were sufficiently sensitive is 0.216. The effect sizes in Study 1 were larger than the minimum effect size.

### Discussion

Study 1 provided preliminary evidence for the relationship between implicit theories and immoral behaviors; that is, ordinary people tended to associate immoral behaviors with an incremental theorist rather than an entity theorist. They believed that a person with an incremental belief on morality would show more immoral behaviors in everyday life and make more immoral decisions in a situation in which immoral behavior offered some advantages. People are able to know others’ views and observe their behaviors in everyday life. When incremental theorists are more likely to engage in immoral behaviors, a large number of observation experiences might form a certain social expectation which was reflected in Study 1. Therefore, based on the results of Study 1, we hypothesize that incremental theory will orient individuals to engage in more immoral behaviors whereas entity theory will not. In Studies 2–4, we focused on whether this correlation originates in the incremental theorists’ actual conduct. Study 2 will further test its stability when the participants are the potential immoral actors instead of merely being observers.

## Study 2

To test our hypothesis, the actor perspective instead of the observer perspective was examined in Study 2. People’s trait-like implicit theories might affect their moral decisions. Holding incremental beliefs might cause individuals to lack internal constraint in the long term, thereby showing more immoral intentions. Implicit theories of morality and a discriminating tendency in a hiring task were measured. We expected that incremental theory would be associated with a higher intention to discriminate.

### Method

#### Participants

Because of the correlational nature of Study 2, a power of 80% and a medium level of *r* (*r* = 0.3) were used to determine the sample size (Cohen, 1992). This computation resulted in a sample size of 84. One hundred and thirty-four participants were recruited online in exchange for ¥6 Yuan (approximately $0.9 USD). Twenty-seven participants were excluded because of not passing the attention test items, leaving a final sample of 107 (44 males and 63 females). The participants’ ages ranged from 21 to 53, with a mean of 29.75 (*SD* = 6.27).

#### Materials and Procedure

The participants were instructed to complete a survey consisting of the measures described below. There were two attention test items that were inserted into the *Implicit Theories of Morality* and *Social Desirability* measurements. The participants were included in the analysis only if they had passed both items. Finally, they were required to report some demographic variables.

##### Implicit theories of morality

The Implicit Theories of Morality Scale was developed by Levy et al. (1998) and was further adapted by Hughes (2015) for the domain of morality. There were eight items to measure both entity (e.g., A person’s moral character is something very basic about them, and it cannot be changed much.) and incremental beliefs (e.g., People can substantially change their moral character.) on morality. The participants were asked to rate the items on a 7-point scale ranging from 1 (*strongly disagree*) to 7 (*strongly agree*). In this measurement, the two implicit theories were mutually exclusive alternatives; thus, a higher score means a stronger inclination toward the incremental belief on morality. The Cronbach’s α was calculated as 0.93.

##### Discrimination intention

A hiring task developed by Kouchaki (2011) was adopted to measure the discrimination intention. We adapted the task to capture the participant’s discrimination intention against carriers of the hepatitis B virus (HBV)^1^. First, the participants were told a cover story that the researchers were hiring a research assistant for an academic institute and that it would be helpful if they could give some opinions. Second, they were provided with the resumes of four applicants. Each applicant was briefly described by grade point average (GPA), research experience, physical status and other basic information. Among them, applicant 3 was the most qualified, that is, the star applicant. He had the highest GPA and 1 year of experience in a research laboratory; however, he was an HBV carrier. Applicant 2 was the next most qualified candidate, that is, the compared applicant. He had a relatively high GPA and 6 months of experience in a research laboratory, and he was healthy. The other two applicants were designed to be less qualified, with lower GPAs and poor work experience. Finally, the participants were asked to vote for these four applicants by distributing 20 votes in total. The degree of HBV discrimination was calculated as the difference between votes for the compared applicant minus votes for the star applicant. The higher the score was, the higher the discrimination intention.

##### Social desirability

As antidiscrimination is a highly socially desired concept, we adopted 20 items (e.g., “I am a completely rational person”) from the Balanced Inventory of Desirable Responding (BIDR; Paulhus, 1991) to control for response bias in the follow-up analysis. The participants were asked to rate the items on a 7-point scale ranging from 1 (*strongly disagree*) to 7 (*strongly agree*). The standard scoring procedure was computed by awarding one point for each response of “6” or “7” and zero points for each response of “5” or less and then summing the points across items. Higher scores showed stronger social desirability. The Cronbach’s α was calculated as 0.85.

### Results

The descriptive statistics are reported in **Table 1**. A linear regression was conducted to explore the effect of implicit theories of morality on the degree of HBV discrimination. The results in **Table 2** indicated that after controlling for the participants’ gender, educational level, family income and social desirability, implicit theories of morality positively predicted the degree of HBV discrimination (β = 0.205, *p* = 0.035, 95% CI [0.05, 1.46]), which means that the greater the tendency toward incremental theory, the more discrimination against HBV carriers in the hiring task there was.

**Table 1:** Descriptive statistics among the key variables in Study 2.

	Implicit theories of morality	Votes for star applicant (A)	Votes for compared applicant (B)	HBV discrimination (B-A)
*M*	3.74	4.28	8.68	4.40
*SD*	1.46	3.27	2.78	5.38


**Table 2:** Regression predicting HBV discrimination (B-A) variables.

Variables		Step 1				Step 2			
		
		*b*		*SE*	β	*b*		*SE*	β
Controlled variables	Gender	-0.782		1.053	-0.072	-1.093		1.045	-0.100
	Educational level	-2.770^†^		1.610	-0.178^†^	-2.879		1.584	-0.185
	Family income	-0.682		0.835	-0.090	-0.802		0.823	-0.106
	Social desirability	-0.004		0.115	-0.004	0.015		0.114	0.014
Independent variable	Implicit theories of morality					0.754^∗^		0.353	0.205^∗^
*F*			1.488				2.143^†^			
Adjusted *R^2^*			0.018				0.051^∗^			
*ΔR^2^*			0.055				0.041^∗^			


*Gender was dummy-coded as 0 for female and 1 for male. Educational level was measured as 1 for high school, 2 for undergraduate, 3 for postgraduate, and 4 for doctorate. ^†^*p* < 0.10, ^∗^*p* < 0.05, ^∗∗^*p* < 0.01, ^∗∗∗^*p* < 0.001.*

### Discussion

In Study 2, we found a positive correlation between incremental moral beliefs and immoral behaviors from the perspective of actors. The stronger the incremental theory one held, the greater the preparedness to engage in discriminating practices. The results of Studies 1 and 2 were in line with our predictions; the findings in the first two studies demonstrated the correlation between incremental beliefs and immoral behaviors, regardless of the participants’ position. However, the causal relationship between implicit beliefs and immoral behaviors was not tested in these studies. In the next two studies, implicit theories were manipulated rather than measured through self-reports as stable and static variables, like in Study 2.

## Study 3

Implicit theories can be manipulated in the laboratory, and doing so might influence people’s moral decisions in subsequent tasks. In Study 3, we primed the two types of implicit theories of morality by requiring participants to read “scientific” articles to replicate the results found in Study 2. We also provided the participants with an altered matrix solving task to examine their immoral behavior. We expected that compared to the participants primed for entity theory, the participants primed for incremental theory would show more cheating behaviors in the matrix solving task.

### Method

#### Participants

An *a priori* power analysis of *t*-tests with a power of 80%, α = 0.05 (one-sided) and a medium effect (*d* = 0.6) was used to determine the sample size, resulting in a sample size of 72. Eighty-five undergraduate and postgraduate students were recruited from the campus online forum; five participants were excluded because they failed to complete the procedure or guessed the purpose of this study. Ultimately, 80 participants (15 males, 65 females) were included in the analysis, comprising 40 participants in the entity condition and the rest in the incremental condition. Their ages ranged from 17 to 28, with a mean of 21.15 (*SD* = 2.53). All participants in this experiment received at least ¥15 Yuan (approximately $2 USD) as a reward.

#### Materials and Procedure

Once the participants entered the laboratory, the experimenter pretended to be leaving for a meeting and asked the participants to read the instructions written on the paper and then complete a series of tasks that ostensibly seemed unrelated with each other. The participants first reported their demographic information and were then instructed to complete a reading task. The participants were randomly divided into two groups (entity condition, incremental condition). The reading task was adapted from Bergen (1991) and was utilized to manipulate implicit theories of morality. In this task, the participants were required to read a scientific article titled “The Origins of Morality: Is the Nature–Nurture Controversy Resolved?,” which looked like an article published in the April 2012 issue of *Psychology Today*. There were two versions of this article; they had the same basic information and expression, but the key opinions about whether morality is stable or malleable were completely opposite. The entity version claimed that morality was genetically determined and could not change over time. For example, one paragraph stated.

John Knowles, the author of the article and a professor at Harvard, concludes that “morality seems to have a very strong genetic component. In addition, the environment seems to play a somewhat important role during the first 3 years of life. After the age of three, though, environmental factors (barring brain damage) seem to have almost no influence on morality.”

By contrast, the incremental version claimed that morality was determined by the environment and could be promoted over time. The paired paragraph in the incremental article stated.

John Knowles, the author of the article and a professor at Harvard, concludes that “morality seems to have a minimal genetic component. People may be born with a given level of morality, but we observe a greatly improved moral level when people enter environments of altruistic and praising morality.”

Both groups of participants were required to answer three questions after reading: (a) to summarize the theme of the article in one sentence, (b) to state the evidence that they thought was the most convincing, and (c) to describe a personal experience in line with the main points of this article. To check the manipulating effect, the participants then completed the *Implicit Theories of Morality Scale* (α = 0.89), which was used in Study 2. Next, the participants were required to complete a matrix solving task adapted from Mazar et al. (2008) to assess the cheating level of the participants. In this task, the participants were presented twenty digital matrices (each based on a set of 12 three-digit numbers) on a computer. They had 15 s per matrix to find two numbers that added up to 10, but there was actually no correct answer^2^. The participants were told that they could obtain ¥10 as a base reward and that for each solved matrix, they could obtain ¥1 as an extra reward. After all matrices had been presented, the participants were instructed to write down the number of solved matrices, take away the corresponding money from a pre-prepared envelope, and then leave the lab. Because there was no correct answer, the self-reported number of solved matrices and the amount of money the participants took away were the representations of cheating. Finally, the participants were debriefed. Three participants suspected that there were no correct answers in the matrix solving task and were thus excluded from the analysis.

### Results

The descriptive statistics are reported in **Table 3**. First, we conducted a chi-square test and independent samples *t*-tests to examine whether the participants in the two groups were homogeneous, finding no differences in gender, age or educational level between the two conditions (*ps* > 0.50). Next, the manipulation check showed a significantly higher score in the Implicit Theories of Morality Scale for the incremental theory group (*M* = 4.53, *SD* = 0.95) than in the entity theory group (*M* = 3.11, *SD* = 1.13; *t* (78) = 6.07, *p* < .001, 95% CI [0.95, 1.88], Cohen’s *d* = 1.360).

**Table 3:** Descriptive statistics for Study 3 variables.

	Gender (number of females)	Age	Education level	Implicit theories measurement	Self-reported number of solved matrices	Amount of money taken away
Incremental condition	33	21.33 ± 2.37	2.30 ± 0.46	4.53 ± 0.95	4.78 ± 5.11	5.40 ± 5.52
Entity condition	32	20.98 ± 2.69	2.25 ± 0.54	3.11 ± 1.13	2.30 ± 2.43	2.28 ± 2.44


*Educational level was measured as 1 for high school, 2 for undergraduate, 3 for postgraduate, and 4 for doctorate.*

Then, an independent samples *t*-test was performed to examine the difference in the self-reported number of solved matrices and the amount of money taken away between the two conditions. The results showed that the participants in the incremental group (*M* = 4.78, *SD* = 5.11) reported a significantly higher number of solved matrices than those in the entity group (*M* = 2.30, *SD* = 2.43; *t*(78) = 2.77, *p* = 0.002, 95% CI [0.68, 4.27], Cohen’s *d* = 0.620). Moreover, the participants in the incremental group (*M* = 5.40, *SD* = 5.52) also took significantly more money away compared to the participants in the entity group (*M* = 2.28, *SD* = 2.44; *t*(78) = 3.28, *p* = 0.002, 95% CI [1.21, 5.04], Cohen’s *d* = 0.731).

### Discussion

In Study 3, a reading task primed the incremental and entity theories of morality. As hypothesized, Study 3 once again showed that compared to entity theory, incremental theory was associated with a higher level of the immoral behavior of cheating.

Study 3, however, had some limitations. First, it lacked a control group; thus, the relative degree of the effect of implicit theories compared to the baseline was unknown. It is possible that the differences between groups were caused by the promoting effect of entity theory on moral behavior rather than the promoting effect of incremental theory on immoral behavior. Second, the entity theory of morality could be distinguished into stable and moral and stable and immoral, and these two sub-entity beliefs might have different impacts on the moral practices with which we were concerned. Stable beliefs on morality enable people to behave consistently with their beliefs (Aquino and Reed, 2002); that is, a stable and moral belief relates to moral behavior, whereas a stable and immoral belief relates to immoral behavior. The effects of the two different beliefs might be offset when placed in one group, which might be a possible reason for the higher immoral behaviors in the incremental group than in the entity group. To eliminate this possibility and to prove the role of entity theory, we distinguish between the two sub-entity beliefs in Study 4. Finally, the manipulation check items have been answered before measuring the dependent variables; therefore, it is reasonable to assume that it was the manipulation check items that made the implicit theories salient. Study 4 attempts to address these weaknesses by adding a blank control group, distinguishing the two types of entity beliefs, and placing the manipulation check in the pilot study.

## Study 4

Study 4 was designed to further examine the association between implicit theories and immoral behaviors and to eliminate the potential competitive explanations noted above. A blank condition was added as a baseline; meanwhile, a new manipulation method was developed to eliminate the possible difference between the two types of entity beliefs (stable and moral, stable and immoral). In Study 4, we first conducted a pilot study to check the effectiveness of the new manipulation method. Then, in the formal study, the participants completed the hiring task (the same as in Study 2) after the implicit theories manipulation. We predicted that the participants in the unstable condition (priming incremental theory) would show more immoral behaviors than those in the two stable conditions and the blank condition and that there would be no differences between the two stable conditions.

### Pilot Study

The purpose of the pilot study was to develop a new manipulation method of implicit theories of morality and to test the effectiveness as well, aiming to distinguish between the two types of entity beliefs.

#### Participants

Because the pilot study consisted of three groups, an *a priori* power analysis of *F*-tests with a power of 80% and an effect size of 0.35 was used to determine the sample size, resulting in a sample size of 84. One hundred and thirty-seven undergraduate students completed an online survey for course credit. Thirteen participants failed to follow the instructions (the content that was written in the manipulation did not correspond to the requirements) and another nine participants failed to pass the attention detection item; thus, their data were excluded. Ultimately, 115 participants (11 males, 104 females) were included in analysis, comprising 43 participants in the stable and moral condition, 36 participants in the stable and immoral condition, and 36 participants in the unstable (incremental) condition. Their ages ranged from 19 to 23, with a mean of 20.39 (*SD* = 0.75).

#### Materials and Procedure

The participants were randomly assigned to one of the three conditions (stable and moral condition, stable and immoral condition, and unstable condition). First, the participants were asked to read a research report that presented the results of a fictitious survey on the moral performance of 2687 college students among 16 universities (including the participants’ university) in Beijing, the capital city of China. In the stable and moral condition, the report concluded that the students from the participants’ school were above average in the moral evaluation, and the participants were then required to write down some moral behaviors typically performed in their university based on their observations. By contrast, in the stable and immoral condition, the report concluded that the students from the participants’ school were below average in the moral evaluation, and the participants were then required to write down some immoral behaviors typically performed in their university based on their observations. In addition, the report of the unstable condition concluded that the students in their school showed an average moral level, and they were required to write down both the moral and immoral behaviors that they observed in their university. The text for each condition was required to be at least 100 words. Participants who did not write matching events as required were excluded, for example, writing irrelevant events or some moral behaviors in the stable and immoral condition. Next, the participants completed two manipulation check items chosen from the *Implicit Theories of Morality Scale* for the relatively high loadings in both Study 2 and Study 3. Item 1 is “People can substantially change their moral character,” and item 2 is “Everyone, no matter who they are, can significantly change their basic moral character” (α = 0.70). The participants were asked to rate the items on a 7-point scale ranging from 1 (*strongly disagree*) to 7 (*strongly agree*). The higher the total score was, the stronger the incremental belief on morality. Additionally, there was an attention detection item, which was same as that in Study 1. Finally, the participants reported their demographic information.

#### Results

The results of the one-way analysis of variance (ANOVA) demonstrated that the main effect of the manipulation was significant [*F*(2,112) = 4.148, *p* = 0.018, η^2^ = 0.069]. *Post hoc* comparisons found that the participants in the unstable condition (*M* = 4.32, *SD* = 1.32) reported higher incremental beliefs than the participants in both the stable and moral condition (*M* = 3.69, *SD* = 1.27; *p* = 0.032, 95% CI [0.06, 1.21]) and the stable and immoral condition (*M* = 3.49, *SD* = 1.28; *p* = 0.007, 95% CI [0.23, 1.43]). In addition, no significant difference between the stable and moral condition (*M* = 3.69, *SD* = 1.27) and the stable and immoral condition (*M* = 3.49, *SD* = 1.28; *p* = 0.493, 95% CI [-0.38, 0.78]) was found.

The pilot study provided a valuable manipulation method of implicit theories of morality that was then used in the formal study.

### Method

#### Participants

Because the formal study consisted of four groups, an *a priori* power analysis of *F*-tests with the same standards in the pilot study was used to determine the sample size, resulting in a sample size of 96. Two hundred and two undergraduate students participated in this study for course credit. They completed all of the tasks in an online system, taking almost 15 min on average. Five participants were excluded because their response time was too long (more than 2 h), and another fourteen participants were excluded due to failing to complete the manipulation task. Ultimately, 183 participants (32 males, 151 females) were included in the analysis, comprising 46 in the stable and moral condition, 35 in the stable and immoral condition, 53 in the unstable (incremental) condition, and 49 in the blank condition. Their ages ranged from 16 to 24, with a mean of 19.92 (*SD* = 1.23).

#### Materials and Procedure

First, the participants were randomly assigned to one of the four conditions (stable and moral condition, stable and immoral condition, unstable condition, and blank condition). Except for those in the blank condition, the participants were primed for corresponding implicit beliefs of morality, as in pilot study. Next, all of the participants completed the hiring task that was used in Study 2 to measure the discrimination level. Finally, the participants reported their demographic information.

### Results

**Table 4** presents the descriptive statistics for the study variables. We conducted ANOVA to test the differences in HBV discrimination among the four conditions. The results showed that there was a significant difference in HBV discrimination among the conditions [*F*(3,179) = 2.86, *p* = 0.038, η^2^ = 0.046]. As hypothesized, the participants in the unstable condition (*M* = 5.21, *SD* = 7.04) showed a higher degree of HBV discrimination than those in the other three conditions (*M*_stable and moral_ = 1.87, *SD* = 5.32, *p* = 0.01, 95% CI [0.82, 5.86]; *M*_stable and immoral_ = 2.43, *SD* = 4.90, *p* = 0.046, 95% CI [0.05, 5.51]; *M*_blank_ = 2.39, *SD* = 7.27, *p* = 0.026, 95% CI [0.34, 5.30]). Furthermore, there was no difference among the other three conditions (*p*s > 0.60).

**Table 4:** Descriptive statistics for Study 4 variables.

Conditions	Votes for star applicant (A)	Votes for compared applicant (B)	HBV discrimination (B-A)
Unstable	5.40 ± 3.43	10.60 ± 4.25	5.21 ± 7.04
Stable and moral	6.83 ± 3.06	8.70 ± 3.08	1.87 ± 5.32
Stable and immoral	5.77 ± 3.22	8.20 ± 2.83	2.43 ± 4.90
Blank	6.63 ± 4.32	9.02 ± 3.92	2.39 ± 7.27


### Discussion

First, by adding a blank control group, Study 4 eliminated the possibility that the hypothesized effect was caused by the promoting effect of entity theory on moral behavior rather than the promoting effect of incremental theory on immoral behavior since the participants in the incremental condition showed the highest degree of HBV discrimination whereas the two entity groups had no difference with the blank group. Second, by decomposing the entity condition into the stable and moral group and the stable and immoral group, Study 4 further clarified one problem; that is, it was not the offset effect of the two sub-entity beliefs that caused the entity theorists to be less likely to engage in immoral behaviors than the incremental theorists since there was no difference in HBV discrimination among the stable and moral group, the stable and immoral group and the blank group.

## General Discussion

People’s implicit theories about human attributes shape the manner in which they understand and react to human actions and outcomes (Dweck et al., 1995a). Evidence from the personality and intelligence research field has suggested that compared to entity theorists (who believe that attributes are fixed, trait-like entities), incremental theorists (who believe that attributes are more dynamic, malleable, and developable) achieve more positive outcomes. The present research found some different evidence and suggested that the incremental theory on morality would lead to immoral behaviors. This effect was shown by using different measures of implicit theories and was captured by a variety of dependent variables. Specifically, we examined how implicit theories of morality with trait-like properties (Studies 1 and 2), primed through a reading task (Study 3) and the perception of the moral performance of one’s group (Study 4) are likely to influence one’s judgment toward the everyday immoral behavior and environmental destruction intention of target people (Study 1), one’s own discrimination against HBV carriers (Studies 2 and 4) and cheating (Study 3). Converging evidence demonstrated that the incremental moral belief (not entity moral belief) increases the tendency toward immoral practice.

Our findings may extend the understanding of the role of implicit theories on moral decisions. First, the findings uncover the dark side of incremental theory to some extent. A large number of previous studies have found many positive aspects of incremental theory, whereas entity theory has often been associated with negative aspects (e.g., Dweck et al., 1995a; Halperin et al., 2011; King, 2012; Cohen-Chen et al., 2014). However, the present research suggests that holding the implicit belief that people’s moral character is malleable might lead to immoral practices. In some areas (such as intelligence, personality), holding developmental and malleable beliefs may be a good thing, but not in the field of morality. Morality, as a social norm, requires a clear and stable standard, which is the basis of moral judgment and behavioral constraints (Barkan et al., 2012). However, for incremental theorists, moral character is dynamic and changeable, and they tend to attribute immoral behavior to context factors, are less likely to make the FAE and forgive immoral practices easily. Thus, they might hold a relatively broader moral standard in moral decisions, which could make them be more likely to behave immorally. By contrast, although entity theory has been considered to link to many negative implications, such as stereotyping, bias, shallow processing of social information, overly rapid interpersonal judgments, and problematic approaches to learning (see the review by Haslam et al., 2006), the present research suggests that it is not always malignant. Since moral character is stable and unchangeable, even small temporary evil will damage the moral self-image of entity theorists, and they tend to avoid behaving immorally. However, the inhibitory effect of entity theory might be limited since the two entity groups did not show less discrimination than the blank group (Study 4). Or, the implication could be that people’s default state (without any priming, just like the blank group in Study 4) of moral theory is much closer to entity theory than to incremental theory. Future research might further examine this issue.

Nevertheless, we have no intention to deny the advantages of incremental beliefs proven in previous studies. For the average person, entity beliefs allow them to have a clear and stable standard that helps them act morally. However, for people who have failed or have not established a moral standard (e.g., prisoners, drug addicts, or growing children), holding incremental beliefs may be a more constructive and positive approach to changing problematic behaviors or promoting moral development. Previous studies have found that people with incremental beliefs made more positive judgments of offender rehabilitation (Leith et al., 2014); children who endorsed sociomoral malleable beliefs were less likely to use aggression to solve conflicts and were more likely to engage in prosocial behavior (Giles and Heyman, 2003); when people realized the plasticity of their own behaviors, they might be more motivated to regulate their moral behavior than average (Vohs and Schooler, 2008). This evidence might suggest that the advantages of incremental theory are embodied in the process of re-establishing norms and promoting moral change and that the benefits of entity theory are embodied in the strengthening of established norms. Future research might continue to focus on the areas of strength of the two theories, which could help the government and educators implement different beliefs in different situations.

Second, the present research also offers a new perspective to explain the relationship between implicit theories and immoral behaviors. We found the association between incremental theory and immoral behaviors from perspectives of both actors and observers. This means that not only are incremental theorists more likely to engage in immoral behaviors, but also that the relationship could be perceived by others. This perception of the potential effect of implicit theories could be as a certain social expectation and, in turn, play a role in an individual’s self-perception and subsequent moral practice. This process is similar to the process of a self-fulfilling prophecy (Berry and Brownlow, 1989; Sinclair et al., 2006) and could be one possible explanations why incremental theorists have a great tendency toward behaving immorally.

Third, the present research suggests that the perception of a group’s moral performance could also affect an individual’s implicit theories of morality (results in Study 4). An individual’s beliefs and attitudes could be affected by one’s group environment (Spencer-Rodgers et al., 2007). Therefore, the stability and fluctuation of the moral performance of the group might shape the individual’s implicit theories of morality. This finding could enrich the priming methodology of implicit theories beyond the traditional reading task that was used. Moreover, it also provides a new perspective to explore the relationship between the characteristics of the group’s and the individual’s implicit theory.

Finally, the limitations of this research and future directions should be considered. First, although we demonstrated that implicit theories have an impact on individuals’ immoral behaviors, the underlying mechanisms were not directly tested. Future research can explore the possible mechanisms noted above, such as moral inference standards, anticipated forgiveness, and moral self-images. Second, although we found that incremental theory increased immoral behaviors in four studies, there was only one scenario in Study 2. Future research could find more connections between trait-like implicit theories and immoral behaviors across various scenarios. Third, the boundary conditions of the dark side of incremental theory should be addressed. Previous studies have found that the ambiguity of the task could influence people’s moral performance; that is, they tend to reduce self-condemnation and show more intention to cheat in a highly ambiguous condition (Effron and Monin, 2010; Brown et al., 2011). In the present study, the measurement of immoral behaviors has a high degree of ambiguity, such as non-direct selection in measuring HBV discrimination and the unsupervised situation in measuring cheating behaviors. Would this phenomenon produce different effects in a low-ambiguity situations? Future research can explore potential moderating factors including the ambiguity of the task. Finally, cross-cultural differences should also be observed by researchers. Research has found that different cultural backgrounds affected people’s tendency toward implicit theories (Morris and Peng, 1994; Dweck et al., 1995b). Would cultural differences affect the tendency of implicit theories of morality and further affect the flexibility of moral rules and even moral compliance in society as a whole? These possibilities will be subject to further exploration.

In conclusion, the present research suggests that implicit theories of morality play an important role in an individual’s moral performance. From the observer perspective, people tend to associate immoral behaviors with incremental theorists rather than entity theorists. From the actor perspective, incremental theorists are more likely to behave immorally than entity theorists. The findings reveal the dark side of incremental theory on morality and enrich the existing research on implicit theories.

## Ethics Statement

This study was carried out in accordance with the recommendations of Human Protection and Ethics Committee of Faculty of Psychology in Beijing Normal University with written informed consent from all subjects. All subjects gave written informed consent in accordance with the Declaration of Helsinki. The protocol was approved by the Human Protection and Ethics Committee of Faculty of Psychology in Beijing Normal University.

## Author Contributions

NH and SZ conceived and designed the study. NH, SZ, and FaW wrote the paper. All authors involved in the research process, discussed results and commented on the manuscript.

## Conflict of Interest Statement

The authors declare that the research was conducted in the absence of any commercial or financial relationships that could be construed as a potential conflict of interest.

## Footnotes

1 Hepatitis B virus (HBV) is not transmitted through contact in everyday life, and the differential treatment of HBV carriers in employment is prohibited by law. However, various types of implicit discriminations still widely exist.

2 In the original vision of this task, participants had 4 min to solve 20 matrices that each had a correct answer. In a pilot study, we found that many participants could solve a majority of the matrices in the given time, and performance was related to participants’ mathematical ability. Given that, we altered the paradigm by setting all of the matrices to have no answers to exclude the effect of mathematical ability. In addition, this is a difficult task for the average person to complete all of the numerical calculations in the matrix within 15 s. Therefore, even if the participants did not find an answer, they rarely doubted that the matrix had no answer.

## APPENDIX

### Full Description of Person A and Person B

*A* and *B* have different opinions about the stability and malleability of morality; their views are as follow:

*A* believes that one’s morality is relatively stable. If someone shows a high moral level in one situation, then he/she will be moral in other similar situations. By contrast, if someone shows a low moral level in one situation, then he/she will be immoral in other similar situations. Hence, *A* believes that we can infer one’s moral level based on one’s behavior. In addition, *A* also believes that one’s morality is inherent and unchangeable. He thinks that our morality has been determined at the earliest of childhood and is mainly determined by genetic factors. Society, education, and some other environmental factors have almost no influence on morality. In other words, “A change in human nature is more difficult than a change in the country.”

*B* believes that one’s morality is relatively changeable. Although someone shows a high moral level in one situation, he/she could be immoral in other similar situations. Similarly, although someone shows a low moral level in one situation, he/she could be moral in other similar situations. Hence, *B* believes that we cannot infer one’s moral level based on one’s behavior. In addition, *B* also believes that people’s morality is malleable and changeable. He thinks that our morality will change with different environments. Society, education, and some other environmental factors have an important influence on morality, but the genetic component seems to have minimal influence. In other words, “Good people who are nearby can make people better, though bad people who are close by can make people worse.”

### Everyday Immoral Activity List

There are a few statements, as below. Please judge who is more likely to perform the following behavior based on the description of Person *A* and Person *B*.

(1) Use unfair means to obtain a better grade on the examination or quiz.
(2) Make up a reason to lie to a friend when temporarily unable to keep an appointment.
(3) Talk about others behind their backs.
(4) Do not give a seat to an elderly person on a bus.
(5) Leave rubbish everywhere.
(6) Find a friend in front of you to jump the queue when waiting.
(7) Have false or plagiarized content in a paper.
(8) Pick up goods in a public place and not return them to the owner.
(9) Show favor to your friends in a judgment or vote.
(10) Use other people’s things without their permission.
